# Vertically aligned InGaN nanowires with engineered axial In composition for highly efficient visible light emission

**DOI:** 10.1038/srep17003

**Published:** 2015-11-20

**Authors:** Mohamed Ebaid, Jin-Ho Kang, Yang-Seok Yoo, Seung-Hyuk Lim, Yong-Hoon Cho, Sang-Wan Ryu

**Affiliations:** 1 Department of Physics, Chonnam National University, Gwangju 500-757, Republic of Korea; 2 Department of Physics and KI for the NanoCentury, Korea Advanced Institute of Science and Technology, Daejeon 305-701, Republic of Korea; 3 Department of Physics, Faculty of Science, Beni-Suef University, Beni-Suef 62511, Egypt

## Abstract

We report on the fabrication of novel InGaN nanowires (NWs) with improved crystalline quality and high radiative efficiency for applications as nanoscale visible light emitters. Pristine InGaN NWs grown under a uniform In/Ga molar flow ratio (UIF) exhibited multi-peak white-like emission and a high density of dislocation-like defects. A phase separation and broad emission with non-uniform luminescent clusters were also observed for a single UIF NW investigated by spatially resolved cathodoluminescence. Hence, we proposed a simple approach based on engineering the axial In content by increasing the In/Ga molar flow ratio at the end of NW growth. This new approach yielded samples with a high luminescence intensity, a narrow emission spectrum, and enhanced crystalline quality. Using time-resolved photoluminescence spectroscopy, the UIF NWs exhibited a long radiative recombination time (τ_r_) and low internal quantum efficiency (IQE) due to strong exciton localization and carrier trapping in defect states. In contrast, NWs with engineered In content demonstrated three times higher IQE and a much shorter τ_r_ due to mitigated In fluctuation and improved crystal quality.

InGaN ternary alloys are of great importance due to their potential to provide tunable emission over most of the solar spectrum using one single crystalline material by simply adjusting the alloy composition^1^. The broad emission produced by these alloys is of particular interest in the development of white light emitting diodes, which can efficiently reduce the required lighting energy that represents a large fraction of the total world energy consumption. The planar InGaN active layers, however, are usually characterized by a high dislocation density that increases with increasing In content, resulting in the well-known green gap^2^. The deterioration of the crystalline quality results in the formation of a large number of non-radiative recombination centres, which can limit the carrier diffusion length and decrease the radiative efficiency^2^,^3^,^4^. Furthermore, the existence of piezoelectric and spontaneous polarization fields in the planar InGaN-based structures can significantly separate the electron and hole wave functions and increase the carrier recombination lifetimes^5^,^6^,^7^. Shrinking the dimensions of the InGaN materials down to the nanoscale in the form of nanowires (NWs) may improve performance through reduced polarization fields^8^, reduced alloying strain^9^, and enhanced In composition^10^. Furthermore, the nanoscale cross-sectional contact between the NWs and the host substrate can effectively reduce the density of threading dislocations and improve the radiative efficiency by reducing the number of non-radiative recombination centers^11^. The light extraction and collection efficiencies can also be improved in the nanostructure geometry due to the significant increase in the surface-to-volume ratio^12^.

Recently, there have been several reports on the synthesis of InGaN NWs by hydride vapour-phase epitaxy (HVPE)^10^ and RF-molecular beam epitaxy (RF-MBE)^13^,^14^; however, the growth of InGaN NWs using metalorganic chemical vapour deposition (MOCVD) has rarely been reported^15^. The growth of InGaN NWs by HVPE is limited by the hydrogen-induced In etching, which can significantly reduce the In content and form unreactive In species^10^. Although catalyst-free InGaN NWs grown by RF-MBE had higher In content, the high cost, slow growth rate, required ultra-high vacuum, and associated surface damage by the RF plasma source make it difficult to commercialize this technique for device applications. Furthermore, extrinsic doping and fabrication of heterojunctions are not available via the HVPE and RF-MBE techniques. In contrast, MOCVD is known for its scalability in the growth of high quality and single crystalline III–V NWs with In contents sufficiently high for light emission in the yellow-green-red spectral ranges^15^. In addition, heteroepitaxy and p-n junction doping are easily achieved using the MOCVD platform, which makes it a promising technique for device applications. In addition, InGaN NWs fabricated by MBE showed a high degree of In-compositional fluctuations that could lead to degradation of the internal quantum efficiency by the associated exciton localization effects^16^. A phase separation in MBE-grown InGaN NWs was also revealed using hard X-ray synchrotron nanoprobe techniques^17^. Recently, it was also reported that MOCVD-grown InGaN NWs are characterized by a multi-peak broad emission resulting from the In-concentration fluctuations^15^. Both In fluctuations and phase separation in InGaN NWs can lead to anisotropic carrier recombination, producing broad spectra with two (or more) emission peaks, typically at the low and high energy sides of the visible spectrum^15^,^17^,^18^.

In this context, we have developed a new approach based on engineering the axial In content during MOCVD growth of InGaN NWs. We have tested several methods, and enhancing the In content at the end of NW growth was found to be the most effective approach to improving the optical emission and morphology of the NWs (supporting information, Fig. S1). By increasing the In/Ga molar flow ratio at the end of NW growth, we could produce a higher In composition and enable the formation of efficient radiative recombination channels to enhance the overall radiative efficiency. The direct growth of band gap-engineered InGaN alloys in the form of nanomaterials could pave the way for the realization of several electronic and optoelectronic devices with reduced degree of complication and low cost.

## Results

In the metal-initiated vapor-liquid-solid (VLS) growth mechanism, the morphology of the starting catalyst (size, shape, and density) plays a crucial role in determining the final morphological and physical properties of the produced NWs. We observed that the morphology of the agglomerated Ni nanoparticles were highly sensitive to the thickness of the initial Ni film. The highest density and dimensional uniformity were achieved using a 0.3 nm Ni film, which is equivalent to two monolayers (ML) of (111) Ni, assuming the film had a face-centred cubic structure and a lattice constant of 0.352 nm. Therefore, one ML of (111) Ni is equivalent to approximately 0.152 nm^19^. The influence of the starting Ni film thickness on the morphology of the nanocatalysts and the corresponding growth of the InGaN NWs can be found in the supporting information, Figs S2 and S3. The role of engineering the axial In composition of vertically aligned InGaN NWs was investigated in their morphological and optical characteristics. The InGaN NWs were grown using Ni-catalysed VLS growth in a vertical MOCVD reactor. Two different structures, with uniform and engineered In/Ga molar flow ratios, were fabricated. Throughout this study, the uniform and engineered In/Ga molar flow ratio samples are referred to as UIF and EIF, respectively.

**Figure 1:**
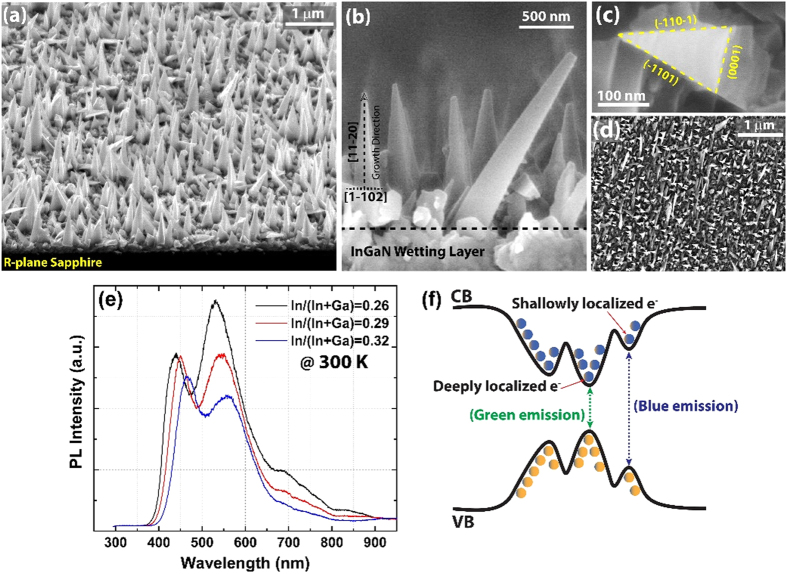
Detailed morphological and optical properties of InGaN NWs. (**a**) Tilted FE-SEM image of the as-grown InGaN NWs on the r-plane sapphire substrate. (**b**) Magnified cross-sectional image for an ensemble of InGaN NWs showing their orientation and their tapered geometry. (**c**) High magnification top-view SEM image showing the triangular cross-section of an InGaN NW. (**d**) Large scale top-view SEM image of the as-grown InGaN NWs with good substrate coverage. (**e**) Room-temperature PL spectra of InGaN NWs as a function of the In/Ga molar flow ratio. (**f**) Schematic illustration of the energy band diagram of UIF NWs showing the shallow and deep localized states and the corresponding blue and green PL emissions.

Figure 1 displays the detailed morphological characteristics of InGaN NWs grown on a r-plane sapphire substrate with the optimized growth parameters of 0.3 nm Ni catalysts, 710 °C, 100 Torr, and 60 min. As shown in Fig. 1(a), InGaN NWs were mostly grown vertically on the r-plane sapphire surface, suggesting that their growth direction was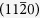, which is the direction normal to the r-plane face of the sapphire substrate^15^,^20^. A magnified cross-sectional image of an ensemble of InGaN NWs is shown in Fig. 1(b). A tapered triangular cross-section with a flat top and an average length of approximately 1 μm can be clearly identified. There is a great difference in the morphology of the InGaN NWs grown by MOCVD and those fabricated by MBE, where the latter are usually shorter than 1 μm and are tapered towards the bottom^13^,^14^. No Ni catalyst was found on the flat top of the InGaN NWs, suggesting a vapour-solid (VS) growth mechanism^21^. A thin film (wetting layer) was unintentionally formed at the bottom of the growth front, which is commonly observed in metal-initiated VLS growth^20^,^21^. The triangular cross-section of the InGaN NWs can also be recognized from the top-view field emission scanning electron microscopy (FE-SEM) image shown in Fig. 1(c). A similar triangular cross-section was observed in GaN NWs grown by the same technique, which may be attributed to the competitive nature of the growth kinetics on different facets^21^,^22^. The three faces of the isosceles triangular cross-section of the InGaN NWs can be indexed as the c-plane for the base and two 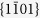 inclined facets, which is consistent with the recent reports of InGaN NWs grown by MOCVD^15^. The high density and the orientation of the InGaN NWs on the substrate are shown in Fig. 1(d), where the bright triangular spots correspond to vertically aligned NWs.

One of the most promising characteristics of InGaN NWs is the shift in the optical emission to longer wavelengths in the visible spectrum, which can be obtained by controlling the In/Ga molar flow ratio. Hence, we investigated the optical emission of the pristine InGaN NWs using room-temperature photoluminescence (PL) as a function of the In/Ga molar flow ratio, as shown in Fig. 1(e). The room-temperature PL spectra collected from an ensemble of InGaN NWs revealed broad white-like emissions extending from approximately 400 to 800 nm. The PL emissions red-shifted with increased In/Ga molar flow ratio, corresponding to enhanced In compositions. All PL spectra were characterized by two emission peaks in the blue and green wavelengths. As shown in the schematic illustration in Fig. 1(f), this emission profile may be attributed to the co-existence of shallow and deep exciton localization states caused by alloy disorder and/or In-concentration fluctuations in the InGaN alloys^18^. The phase separation in the InGaN alloy caused by the immiscibility of GaN and InN was also reported to contribute to these emission characteristics^23^. Consequently, the blue and green emission peaks in the PL spectra can be attributed to radiative recombination in the shallow (low In-content) and deep (high In-content) localization states, respectively.

**Figure 2:**
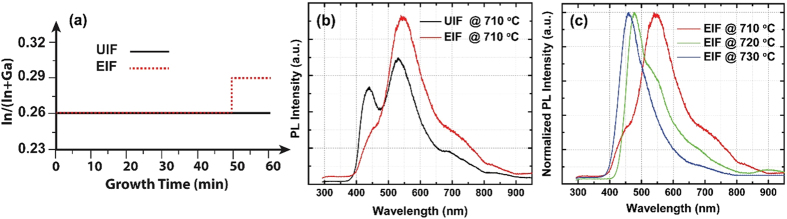
(**a**) Schematic illustrating the In-flow modulation procedure during the growth of InGaN NWs by MOCVD. (**b**) Room-temperature PL measurements of the UIF and the EIF InGaN NWs. (**c**) Tunable emission of the EIF NWs as a function of growth temperature.

To improve the optical characteristics of the InGaN NWs, we proposed a simple approach to engineer the axial In content by modulating the In/Ga molar flow ratio during MOCVD growth. To avoid In-concentration fluctuations appeared in the UIF samples, the trimethylindium (TMIn) flow rate was only increased during the last 10 min of the overall growth time (60 min). The TMIn flow rate was increased from 1.9 to 2.3 μmol/min, which corresponds to In/Ga molar flow ratios of 0.26 and 0.29, respectively (Fig. 2(a)). As shown in Fig. 2(b), the room-temperature PL measurements revealed a prominent enhancement in the optical emission of the EIF InGaN NWs compared to that of the UIF samples. A large increase in the ratio between the green and blue emissions was observed for the EIF NWs. By controlling the temperature over the entire growth time of the EIF NWs, the PL emission could be tuned from the blue to the green wavelengths as a result of the In-content variation (Fig. 2(c)). As the PL emission was blue-shifted, the spectrum was characterized by an intense emission peak, signifying that the In-concentration fluctuation was the dominant parameter affecting the optical emission of the InGaN NWs. Achieving a widely tunable emission from blue to green wavelengths using one single crystal NW structure is highly desirable and emphasizes the value of InGaN NWs as emerging nanoscale visible light emitters. The tunable emission coupled with intense narrow peaks achieved in this study is superior to that of similar InGaN NWs grown by MOCVD, which were characterized by a very large full-width at half-maximum and multi-peak emissions^15^.

**Figure 3:**
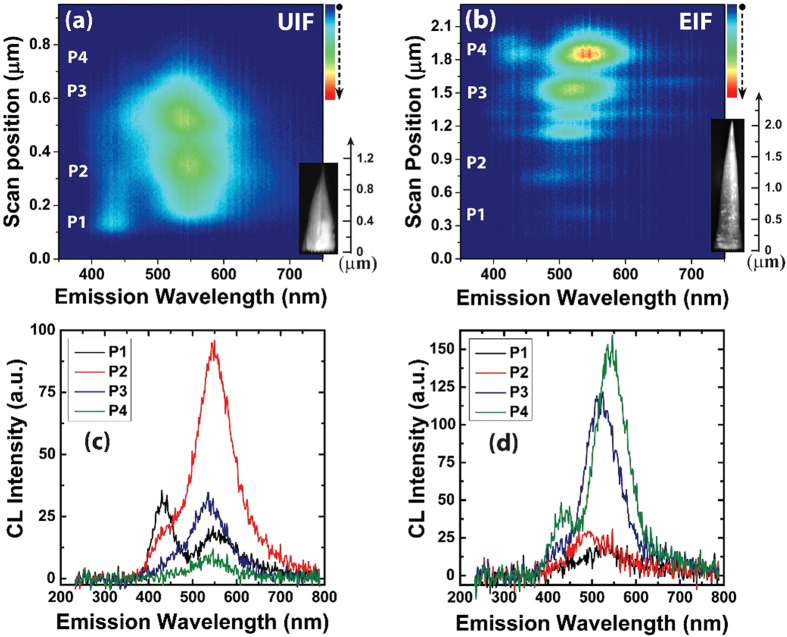
CL line-scan mapping of single InGaN NWs: (**a**) UIF and (**b**) EIF. (**c**,**d**) Resolved CL spectra taken from several spots in the line-scan mapping of the investigated InGaN NWs shown in (**a**,**b**), respectively.

To investigate the local optical emission as a function of the axial In composition of the EIF InGaN NWs and compare it to that of the UIF NWs, we performed spatially resolved cathodoluminescence (CL) line-scan mapping on single NWs of each type. Investigating the optical emission of individual InGaN NWs can provide meaningful insight into the emission mechanism, where contributions from the wetting layer, as well as morphological non-uniformities within NW ensembles, can be eliminated. The two types of InGaN NWs produced different emission profiles, suggesting distinct carrier recombination mechanisms. While the EIF InGaN NWs exhibited a narrow emission spectrum, whose intensity increased progressively towards the high In-content top portion, a broad emission with inhomogeneous luminescent clusters was exhibited by the UIF NWs, as shown in Fig. 3(a,b). The CL emission of the EIF NWs was slightly red-shifted towards the tip of the NW, indicating enhanced In composition. The CL line-scan mappings for both samples can be more directly compared through the resolved spectra shown in Fig. 3(c,d), which were chosen from comparable points along the axes of the investigated NWs. The UIF NW appeared to have an inhomogeneous axial In composition (In-concentration fluctuations), indicated by its broad spectrum consisting of different luminescent clusters. Furthermore, the appearance of two different emissions (blue and green) may also imply that this NW underwent phase separation (low In-content and high In-content phases), which is consistent with the room-temperature PL in Fig. 1(e). A similar phase separation was detected for a single InGaN NW grown by MBE on a bare n-type Si wafer using nano-X-ray fluorescence mapping^17^, suggesting this may be a general problem governing the growth of InGaN NWs with different techniques and on different substrates. In this study, the phase separation and fluctuations in the In composition were significantly reduced by controlling the axial In composition.

**Figure 4:**
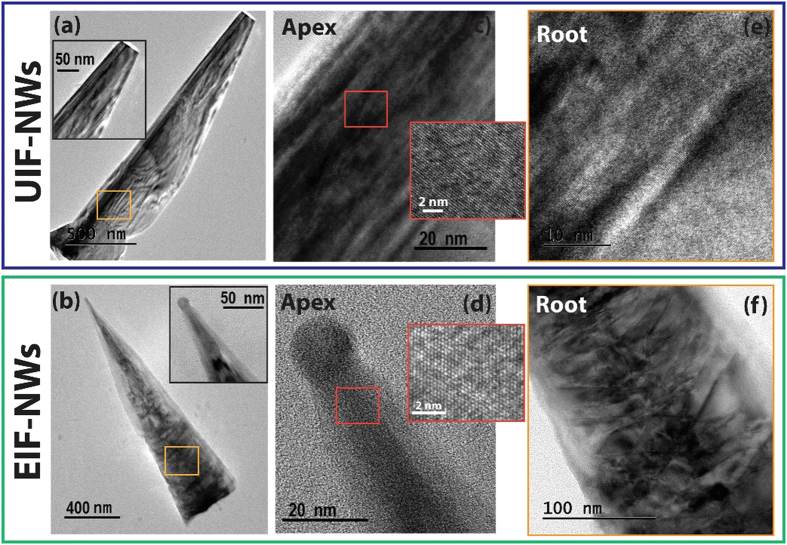
Microstructural characteristics of UIF and EIF NWs from TEM imaging. (**a**,**b**) BF-TEM images of the investigated UIF and EIF InGaN NWs, respectively. (**c**,**d**) High resolution TEM images taken selectively at the apex of each NW. The insets are lattice-resolved TEM images showing the crystal quality at the atomic scale. (**e**,**f**) High resolution TEM images taken at the roots of the investigated NWs, which are highlighted with the orange squares in (**a**,**b**).

The microstructure of the UIF and EIF InGaN NWs, studied by bright field transmission electron microscopy (BF-TEM), also revealed dramatic morphological differences between the two types of NWs. Both InGaN NW types were tapered towards their tips, with the EIF NW showing stronger tapering. The UIF NWs were characterized by a flat top with no metal catalyst, while the EIF NWs exhibited a very sharp tip with metal catalyst remains. These morphological features suggest a transition in the growth mode from (vapor-solid) VS to VLS for the UIF and EIF NWs, respectively. This change in the growth mode to VLS for EIF may be attributed to the expanded liquid phase domain of the metal catalyst through the absorption of more In atoms at the end of NW growth^21^,^24^. It was reported that the vertical growth rate of the VLS grown NWs can be significantly enhanced by adding In-metal to the starting catalyst^21^ or by the flow of In-based vapor (In-surfactant) during the MOCVD growth^24^. Both approaches suggested that the melting point of the metal catalyst will be reduced by the incorporation of In atoms, which will expand the domain of the liquid phase of the metal catalyst and increase the number of absorbed ad-atoms. Consequently, the metal catalyst can be supersaturated faster and more atoms can participate in the formation of the NW. On the other hand, since the Ni-catalyst was used to initiate the growth of both types of InGaN NWs, we believe that the disappearance of the metal in the case of the UIF NWs (VS growth mode) is due to the consumption of this catalyst and/or its incomplete liquefaction during the growth^25^. Apparently, the UIF InGaN NWs suffered from a high density of dislocation-like defects along their entire axes; in contrast, the crystalline quality progressively improved towards the apex of the EIF NWs, where the In content is expected to be higher. The lattice-resolved TEM images, shown in the insets of Fig. 4(c,d), confirm the superior crystalline quality of the EIF NWs, which are characterized by a highly ordered single crystal structure. The roots of both types of InGaN NWs exhibited a high density of defects, suggesting that the enhanced In composition at the top of the EIF is the dominant factor in enhancing the crystalline quality.

**Figure 5:**
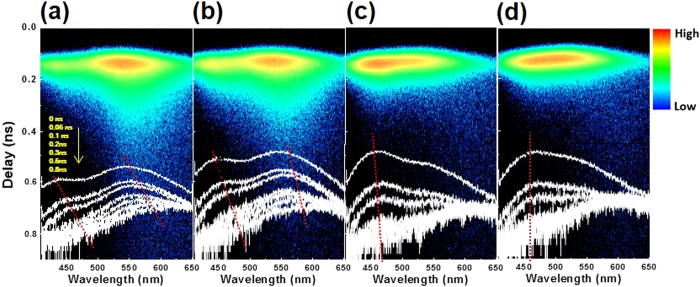
Time-dependent PL decay represented by streak camera three-dimensional mapping images that include the time evolution of the PL emission. (**a**) UIF reference sample grown at 710 °C. (**b**) EIF sample grown at 710 °C. (**c**) EIF sample grown at 720 °C. (**d**) EIF sample grown at 730 °C.

To gain insight into the influence of engineering the axial In composition on the optical characteristics of InGaN NWs, the carrier recombination dynamics were investigated using time-resolved PL (TRPL) spectroscopy. The carrier dynamics were studied for the UIF InGaN NWs as the reference sample and for EIF NWs grown at different temperatures. Based on the room temperature PL spectra shown in Fig. 2, the pumping wavelength of the Ti:sapphire pulsed laser was adjusted to 380 nm to adequately analyze the whole luminescence spectrum. Depending on the sample growth conditions, distinct recombination dynamics were observed in the time-resolved streak camera images, shown in Fig. 5, and in the time evolution of the PL emission, depicted at the bottom of each image. Both UIF and EIF samples grown at 710 °C exhibited two-peak decay, i.e., high energy (blue) and low energy (green) decays. This behaviour can be attributed to the spatial fluctuation of In concentration along the axis of the InGaN NWs, which can lead to the formation of shallow (high energy) and deep (low energy) recombination centers^26^,^27^,^28^. The carrier recombination in the UIF sample mostly occurred through the deeply localized states, which showed a higher PL emission intensity than the shallow localized states, suggesting a high degree of In fluctuation. The PL intensity of the shallow localized states increased in the EIF samples with increasing growth temperature, and at 730 °C, the carriers mostly recombined through the shallowly localized states. Furthermore, a spectral red-shift was observed in the time evolution of the PL emission, which was negligible in the case of the EIF sample grown at 730 °C. This time-dependent spectral red-shift can be attributed to the carrier recombination through the lowest energy states (deeply localized states)^27^. Following the excitation pulse, the carrier density was very high and recombination in the shallow and deep localized states could be observed. However, the number of photogenerated carriers significantly reduced with time, and only the recombination in the deeply localized states was observable, leading to the red-shift of the PL emission. Together, the PL decay behaviour and time-dependent spectral red-shift are typically attributed to an exciton localization mechanism caused by the In-concentration fluctuation in InGaN NWs.

**Figure 6:**
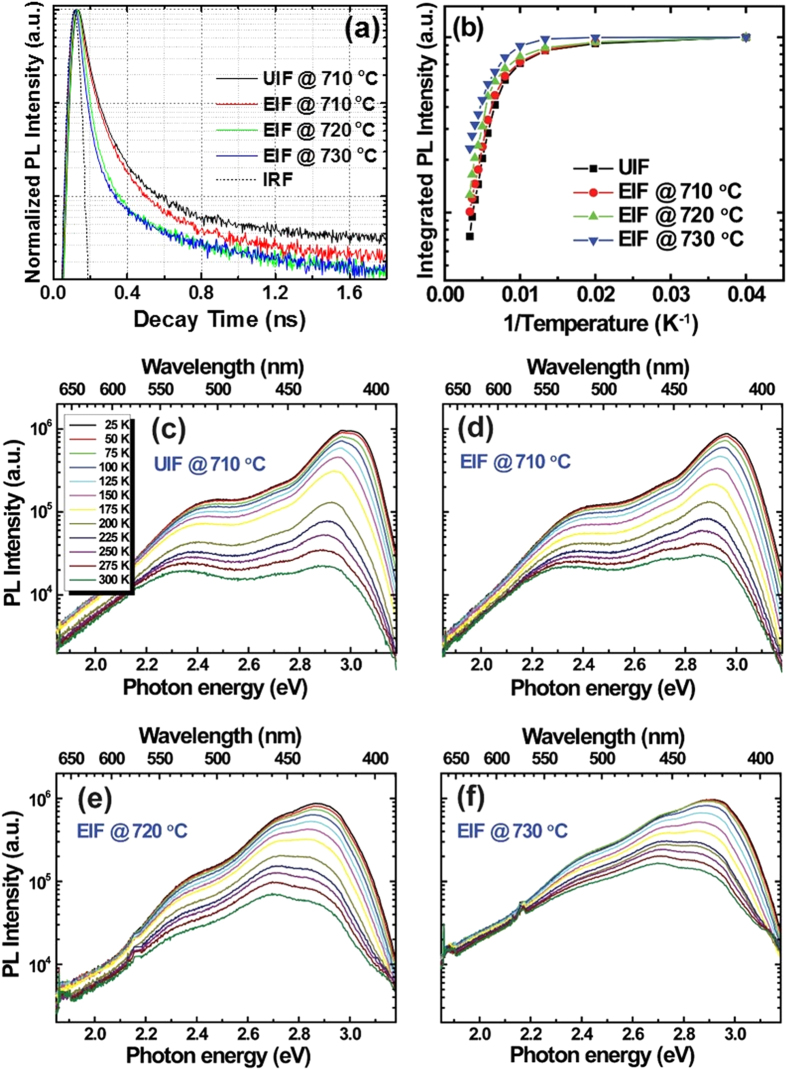
(**a**) TRPL spectra of the same samples measured in Fig. 4.8. (**b**) IQE of InGaN NW samples, as measured from the Arrhenius plots of the temperature-dependent variations in the integrated PL intensity. Temperature-dependent PL for (**c**) UIF reference sample grown at 710 °C, (**d**) EIF sample grown at 710 °C, (**e**) EIF sample grown at 720 °C, and (**f**) EIF sample grown at 730 °C.

To further confirm the effects of exciton localization on the carrier recombination dynamics for both types of InGaN NWs and to calculate the radiative and non-radiative carrier lifetime components, temperature-dependent PL was conducted. First, the TRPL decay curves, for the same samples shown in Fig. 5, were taken and normalized for direct comparison (Fig. 6(a)). All samples exhibited a non-exponential decay profile, which is commonly observed in InGaN-based structures due to spatial In concentration non-uniformity^26^,^27^,^29^. The carrier lifetimes could be determined using TRPL and temperature-dependent PL measurements. Initially, the decay time (τ) was simply calculated as the time at which the value of the PL intensity was reduced by the factor 1/e from its maximum value^28^. Then, using its relationships with the internal quantum efficiency (IQE) and τ, the radiative (τ_r_) and non-radiative (τ_nr_) carrier lifetime components could be calculated. The IQE was measured from the Arrhenius plot representing the variation of the normalized integrated PL intensity with temperature, as shown in Fig. 6(b). The PL peak intensity exhibited saturation up to 100 K and then reduced with increasing measuring temperature. The UIF sample exhibited the lowest IQE of approximately 7.3%, while the value of the IQE was up to three times higher (23%) in the EIF sample grown at 730 °C. Greater IQE values for the EIF samples imply that the radiative recombination was more efficient in these NWs than in the UIF sample, which can be attributed to the enhanced crystalline quality in the former^26^,^28^. It is worth mentioning that the IQE determined in the EIF sample grown at 730 °C is approximately two times higher than that of similar InGaN NWs grown by MBE^16^. After determining the values of the IQE and τ, we calculated the τ_r_ and τ_nr_ carrier lifetimes using the relations 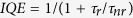 and 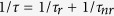, which yield 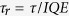. All parameters extracted from these calculations are listed in Table 1. The results revealed that while τ_nr_ changed slightly, τ_r_ significantly decreased in the EIF InGaN NW samples, reaching 290 ps at 730 °C, compared to 1.7 ns in the UIF case. The longer decay time in the UIF sample can be explained by the strong exciton localization in the In-rich clusters and carrier trapping by the defect states^28^. The temperature-dependent variation of the PL shown in Fig. 6(c–f) revealed different carrier localization effects with respect to the sample type. The conventional S-shaped variation of the PL peak energy was observed for the UIF sample, suggesting strong exciton localization effects. This S-shaped behaviour was significantly reduced in the EIF samples and practically vanished in the sample grown at 730 °C (see supporting information, Fig. S4). Hence, the shorter carrier lifetimes observed in the EIF samples can be attributed to the reduced exciton localization effects due smaller In fluctuations and enhanced crystalline quality. These findings emphasize that the carrier recombination dynamics in InGaN NWs are governed by the crystalline quality and the exciton localization effects, which can be controlled by engineering the axial In composition. Reduced exciton localization was the dominant factor in improving the optical quality of the EIF NWs.

## Discussion

In an attempt to more thoroughly understand the physical properties of the emerging InGaN NWs, we investigated the growth and carrier recombination dynamics using different microscopic and spectroscopic techniques. InGaN NWs grown with UIF contained a high density of dislocation-like defects and a high degree of In-compositional fluctuations, which led to poor optical and crystalline qualities. By increasing the In/Ga molar flow ratio during the last 10 min of growth (total growth time of 60 min), a significant improvement in the optical and morphological characteristics was observed. CL line-scan mapping revealed a clear enhancement in the luminescence of EIF NWs, which showed a narrow spectrum with increased intensity at the top of the NWs. The crystal quality was also improved by controlling the axial In composition in the InGaN NWs, leading to a highly ordered InGaN crystal, as observed by lattice-resolved TEM images. These differences between the UIF and EIF NWs were well supported by the TRPL and temperature-dependent PL measurements. Because of the high degree of In fluctuation and poor crystalline quality, the carrier lifetime in the UIF NWs was approximately six times longer than that in the EIF NWs due to the associated trapping and localization effects. Compared to the InGaN NWs grown under UIF or even the InGaN quantum well structures, the EIF NWs possessed superior characteristics, including higher crystalline quality, higher density of radiative recombination centres, and shorter radiative recombination lifetimes. These observations lead to a more thorough understanding of the physical properties of the emerging InGaN NWs for applications in novel visible light emitters and solar light harvesting applications.

## Methods

### MOCVD Growth

A high density of vertically aligned InGaN NWs was synthesized on a 2-inch r-plane sapphire substrate via Ni-catalysed VLS growth in a vertical cold-wall MOCVD reactor. The starting Ni film thickness was varied, and an ultra-thin film of approximately two Ni mono-layers (~0.3 nm) yielded the best morphological and optical properties. The ultra-thin Ni metal film was first deposited on the bare r-plane sapphire substrate by e-beam evaporation, which was subsequently loaded into the MOCVD reactor, where it was annealed at 960 °C and 100 Torr for 10 min in a H_2_ environment to form nanoscale Ni particles. Immediately after Ni agglomeration, the reactor temperature was reduced to 710 °C, and N_2_ was used as the carrier gas to avoid any H_2_-induced In etching during the growth of the InGaN NWs. NH_3_, trimethylgallium (TMGa), and TMIn were introduced to the reactor as the N, Ga, and In precursors, respectively. For the UIF sample, the flow rates of NH_3_, TMGa, and TMIn were fixed at 67 mmol/min, 4.6 μmol/min, and 1.9 μmol/min, respectively; however, the TMIn flow rate was increased in the case of the EIF sample to 2.3 μmol/min at the end of the growth. The reactor pressure was maintained at 100 Torr during the growth of InGaN NWs, which continued for 60 min.

### Morphology and microstructure

The morphological evolution of the as-grown InGaN NWs was measured using FE-SEM (JSM-6700F, JEOL Japan). The microstructure and crystal quality at the atomic resolution were investigated using high resolution BF-TEM (JEM-2100F, JEOL Japan).

### Photoluminescence spectroscopy

The room temperature PL was measured on ensembles of InGaN NWs using a 266 nm diode-pumped solid-state (DPSS) laser with an average optical power of 30 mW at the sample surface. The carrier dynamics of the UIF and EIF samples were studied via TRPL using a Ti:sapphire pulsed laser with a small optical power of approximately 10 μW. The PL decays were detected by a streak camera (Hamamatsu, C7700-01) with a time resolution lower than 300 fs and a repetition rate of approximately 4 MHz.

### Cathodoluminescence spectroscopy

A spatially resolved CL system (Gatan, Mono CL4) with an accelerating voltage of 3 keV and a step size of approximately 2 nm was used to study the local emission of single InGaN NWs. To investigate the spatially resolved CL, as-grown InGaN NWs were detached from the sapphire substrate and dispersed on a cleaned Si wafer, where they were precisely selected and analysed.

## Additional Information

**How to cite this article**: Ebaid, M. *et al.* Vertically aligned InGaN nanowires with engineered axial In composition for highly efficient visible light emission. *Sci. Rep.* **5**, 17003; doi: 10.1038/srep17003 (2015).

## Supplementary Material

Supplementary Information

## Figures and Tables

**Table 1:** The measured parameters from TRPL and temperature-dependent PL for the UIF reference sample and EIF samples.

Sample	τ (ns)	IQE (%)	τ_r_ (ns)	τ_nr_ (ns)
UIF @ 710 °C	0.121	7.3	1.7	0.130
EIF @ 710 °C	0.111	10	1.1	0.123
EIF @ 720 °C	0.072	13	0.56	0.083
EIF @ 730 °C	0.066	23	0.29	0.085

The TRPL data were measured at room temperature.

## References

[b1] Kuykendall T., Ulrich P., Aloni S. & Yang P. Complete composition tunability of InGaN nanowires using a combinatorial approach. Nat. Mater. 6, 951–956 (2007). 17965718 10.1038/nmat2037

[b2] Park I. K. & Park S. J. Green Gap Spectral Range Light-Emitting Diodes with Self-Assembled InGaN Quantum Dots Formed by Enhanced Phase Separation. Appl. Phys. Express. 4, 042102 (2011).

[b3] Cherns D., Henley S. J. & Ponce F. A. Edge and screw dislocations as nonradiative centers in InGaN/GaN quantum well luminescence. Appl. Phys. Lett. 78, 2691–2693 (2011).

[b4] Speck J. S. & Rosner S. J. The role of threading dislocations in the physical properties of GaN and its alloys. Physica B. 273-274, 24–32 (1999).

[b5] Arif R. A., Ee Y. K. & Tansu N. Polarization engineering via staggered InGaN quantum wells for radiative efficiency enhancement of light emitting diodes. Appl. Phys. Lett. 91, 091110–091113 (2007).

[b6] Renner F. *et al.* Quantitative analysis of the polarization fields and absorption changes in InGaN/GaN quantum wells with electroabsorption spectroscopy. Appl. Phys. Lett. 81, 490–492 (2002).

[b7] Wagner J., Ramakrishnan A., Obloh H. & Maier M. Effect of strain and associated piezoelectric fields in InGaN/GaN quantum wells probed by resonant Raman scattering. Appl. Phys. Lett. 74, 3863–3865 (1999).

[b8] Xu H. *et al.* Polarization control in GaN nanowire lasers. Opt. Express. 22, 19198 (2014). 25321005 10.1364/OE.22.019198

[b9] Xiang H. J., Wei S. H., Da Silva J. L. F. & Li J. B. Strain Relaxation and Band-Gap Tunability in Ternary InxGa1-xN Nanowires. Phys. Rev. B. 78, 193301 (2008).

[b10] Hwa-Mok K. *et al.* Formation of InGaN Nanorods with Indium Mole Fractions by Hydride Vapor Phase Epitaxy. Phys. Status Solidi B. 241, 2802–2805 (2004).

[b11] Stoica T. *et al.* Interface and Wetting Layer Effect on the Catalyst-Free Nucleation and Growth of GaN Nanowires. Small. 4, 751–754 (2008). 18535990 10.1002/smll.200700936

[b12] Jiang B. *et al.* Effects of reduced exciton diffusion in InGaN/GaN multiple quantum well nanorods. Opt. Express. 20, 13478 (2012). 22714375 10.1364/OE.20.013478

[b13] Wu K. M., Pan Y. & Liu C. InGaN nanorod arrays grown by molecular beam epitaxy: Growth mechanism structural and optical properties. Appl. Surf. Sci. 255, 6705–6709 (2009).

[b14] Tabata T., Paek J., Honda Y., Yamaguchi M. & Amano H. Growth of InGaN nanowires on a (111) Si substrate by RF-MBE. Phys. Status Solidi C. 9, 3–4 (2012).

[b15] Kuo H. C., Oh T. S. & Ku P. C. MOCVD growth of vertically aligned InGaN nanowires. J. Cryst. Growth. 370, 311–313 (2013).

[b16] Murotani H. *et al.* Effects of exciton localization on internal quantum efficiency of InGaN nanowires. J. Appl. Phys. 114, 153506 (2013).

[b17] Ruiz J. S., Criado G. M., Denker C., Malindretos J. & Rizzi A. Phase Separation in Single InxGa1−xN Nanowires Revealed through a Hard X‑ray Synchrotron Nanoprobe. Nano Lett. 14, 1300–1305 (2014). 24502255 10.1021/nl4042752

[b18] You G. *et al.* Excitation dependent two-component spontaneous emission and ultrafast amplified spontaneous emission in dislocation-free InGaN nanowires. Appl. Phys. Lett. 102, 091105 (2013).

[b19] Li Q. & Wang G. T. Improvement in aligned GaN nanowire growth using submonolayer Ni catalyst films. Appl. Phys. Lett. 93, 043119 (2008).

[b20] Kuykendall T. R., Altoe M. V. P., Ogletree D. F. & Aloni S. Catalyst-Directed Crystallographic Orientation Control of GaN Nanowire Growth. Nano Lett. 14, 6767–6773 (2014). 25390285 10.1021/nl502079v

[b21] Ebaid M., Kang J. H., Lee J. K. & Ryu S. W. Controlled growth mode of high-aspect-ratio GaN nanorods by Ni/In/Ga catalyst. J. Phys. D: Appl. Phys. 46, 385105–385112 (2013).

[b22] Kuykendall T. *et al.* Metalorganic chemical vapor deposition route to GaN nanowires with triangular cross sections. Nano Lett. 3, 1063 (2003).

[b23] Lai Y. L. *et al.* Origins of efficient green light emission in phase-separated InGaN quantum wells. Nanotech. 17, 3734–3739 (2006).

[b24] Su J. *et al.* Catalytic growth of group III-nitride nanowires and nanostructures by metalorganic chemical vapor deposition. Appl. Phys. Lett. 86, 013105 (2005).

[b25] Purushothaman V., Ramakrishnan V. & Jeganathan K. Interplay of VLS and VS growth mechanism for GaN nanowires by a selfcatalytic approach. RSC Adv. 2, 4802–4806 (2012).

[b26] Ebaid M. *et al.* Ultrashort carrier lifetime of vapor–liquid–solid-grown GaN/InGaN multi-quantum-well coaxial nanorods. Acta Materialia. 65, 118–124 (2014).

[b27] Kim J. H. *et al.* Toward highly radiative white light emitting nanostructures: a new approach to dislocation eliminated GaN/InGaN core–shell nanostructures with a negligible polarization field. Nanoscale. 6, 14213–14220 (2014). 25225912 10.1039/c4nr03365e

[b28] Jeong H. *et al.* Carrier localization in In-rich InGaN/GaN multiple quantum wells for green light-emitting diodes. Sci. Rep. 5, 9373 (2015). 25792246 10.1038/srep09373 PMC4366764

[b29] Ebaid M., Kang J. H., Lim S. H., Cho Y. H. & Ryu S. W. Towards highly efficient photoanodes: the role of carrier dynamics on the photoelectrochemical performance of InGaN/GaN multiple quantum well coaxial nanowires. RSC Adv. 5, 23303–23310 (2015).

