# Supplementary material for: Vertically aligned InGaN nanowires with engineered axial In composition for highly efficient visible light emission
Source: Sci Rep. 2015 Nov 20;5:17003. doi: 10.1038/srep17003 (PMC4653627; doi:10.1038/srep17003)
Supplement: Supplementary Information [file srep17003-s1.pdf]

Supporting Information

Vertically aligned InGaN nanowires with engineered axial In composition for highly efficient visible light emission

Mohamed Ebaid[1,3], Jin-Ho Kang[1], Yang-Seok Yoo[2], Seung-Hyuk Lim[2], Yong-Hoon Cho[2], Sang-Wan Ryu[1,*]

[1]Department of Physics, Chonnam National University, Gwangju 500-757, Republic of Korea
[2]Department of Physics and KI for the NanoCentury, Korea Advanced Institute of Science and Technology, Daejeon 305-701, Republic of Korea
[3]Department of Physics, Faculty of Science, Beni-Suef University, Beni-Suef 62511, Egypt

*Corresponding author: S.W.R (sangwan@chonnam.ac.kr)

S1: *The optimization of composition-engineered InGaN NWs:*

To optimize the engineering of In content in the InGaN NWs, we tested several procedures, as shown in Fig. S1 (a). The approach was based on increasing the In/Ga molar flow ratio at the end of growth; however, the duration over which this ratio was increased was critical. We found that increasing the TMIn flow rate for more than 10 min caused severe degradation of the InGaN NW morphology. As shown schematically in Fig. S1 (b), the aspect ratio, as well as the symmetry of the EIF NWs, degraded for durations of increased flow longer than 10 min. The SEM images, taken from different viewing angles, shown in Fig. S1 (d-g), confirmed this trend. The optical properties as a function of the axial In-content profile were further measured to determine the optimization of our structures. As shown in Fig. S1 (c), the highest photoluminescence (PL) intensity and the narrowest peak were achieved when the time was only 10 min. As the time ratio between the low and high TMIn flow rates increased, significant degradation of the optical emission was observed. Based on these measurements, we increased the TMIn flow rate for only 10 min during the total growth time of the NWs (60 min).

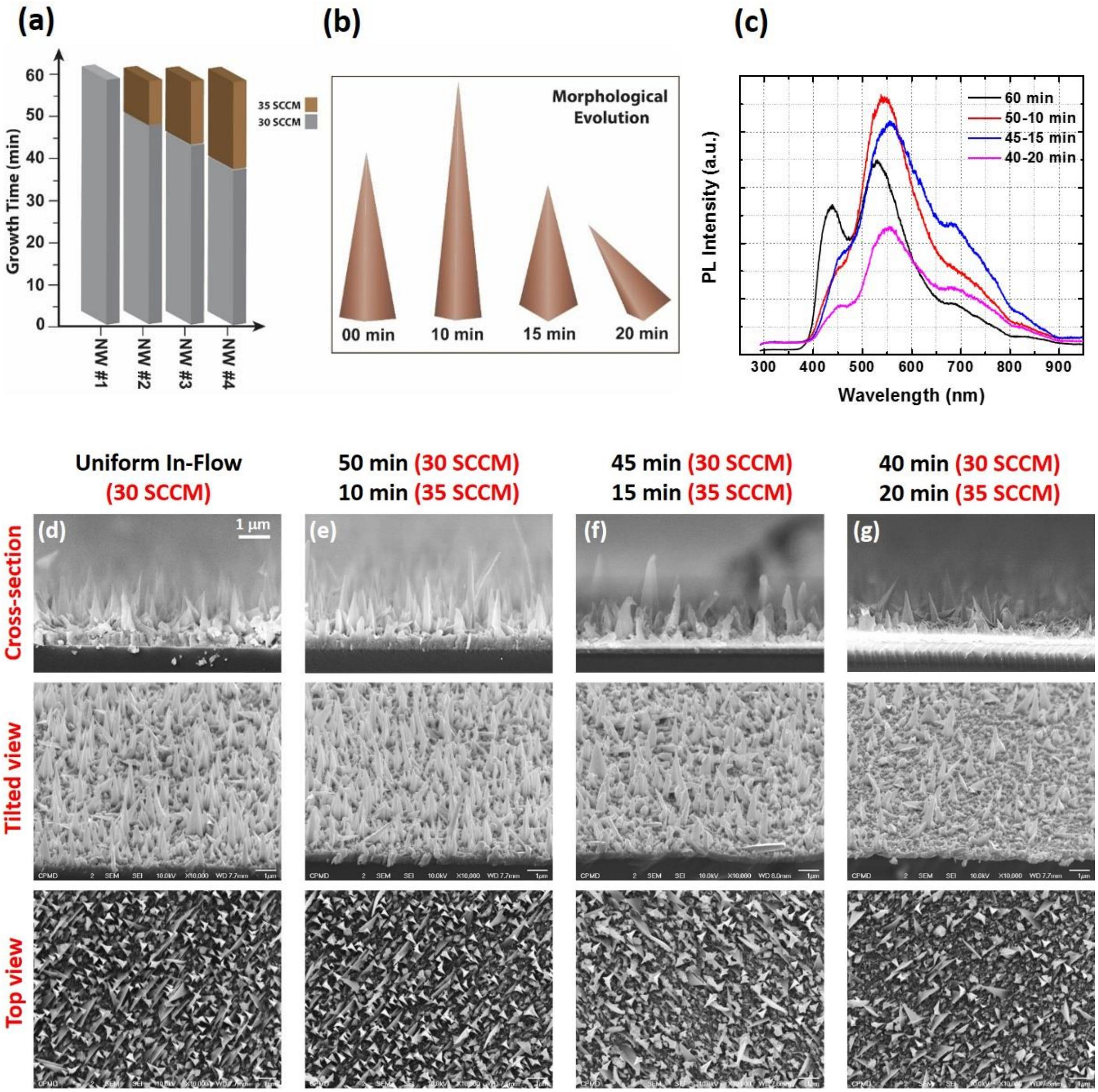

Figure S1. The optimization of the In-flow parameters for the growth of InGaN NWs. (a-b) Schematic diagrams showing the time ratio between the low and high TMIn flow rates and the corresponding morphological changes, respectively. (c) PL spectra taken as a function of the time ratio between the low and high TMIn flow rates. (d-g) SEM images showing the modification of the NW morphology at different viewing angles.

S2: *The morphology of the agglomerated Ni nanoparticles after annealing*

In the metal-initiated VLS growth mechanism, the nature of the starting catalyst (size, shape, and density) plays a crucial role in the final morphological and physical properties of the produced NWs. A thin Ni metal film was first deposited on the bare r-plane sapphire substrate by e-beam evaporation and subsequently loaded into the MOCVD reactor, where it was annealed at 960 ˚C and 100 Torr for 10 min in a H_2 environment to form nanoscale Ni particles. We observed that the geometry of the agglomerated Ni nanoparticles was highly sensitive to the thickness of the initial Ni film. As shown in Fig. S2, the highest density and the greatest uniformity were achieved using a 0.3 nm Ni film, which is equivalent to two monolayers (ML) of (111) Ni. The uniformity of the size and shape of the Ni nanocatalysts degraded with increasing Ni film thickness, which can be observed from the size distribution histograms measured by ImageJ software and displayed as insets in Fig. S2.

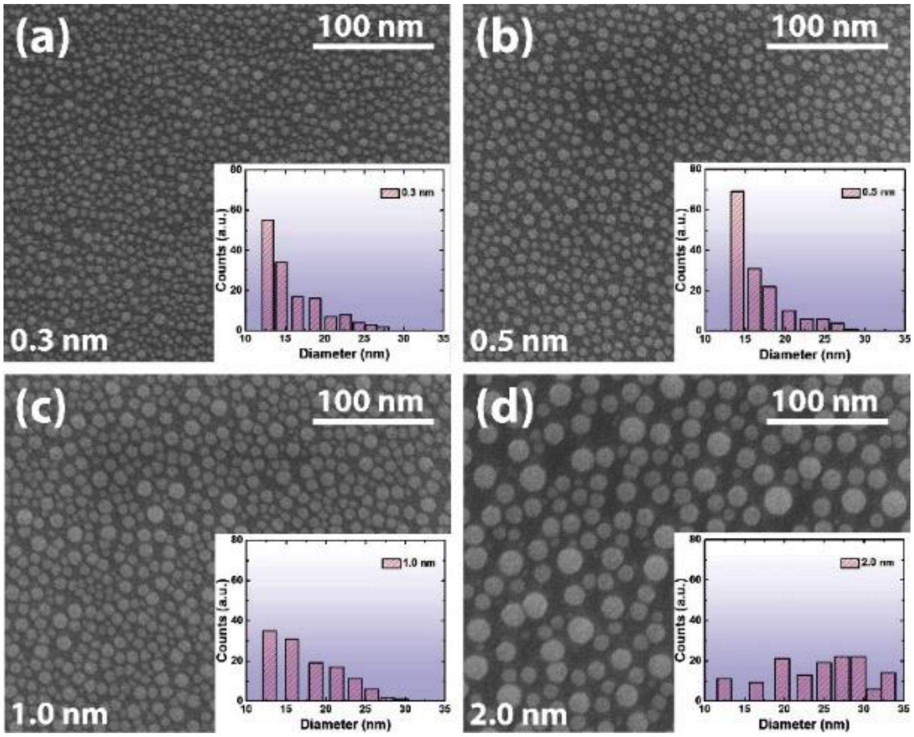

Figure S2. Typical FE-SEM images illustrating the effect of the starting Ni film thickness on the size and density of the agglomerated Ni nanoparticles: (a) 0.3 nm; (b) 0.5 nm; (c) 1.0 nm; (d) 2.0 nm.

S3: *The dependence of NW morphology on the Ni catalyst thickness:*

Under the same growth conditions, but for different Ni film thicknesses, InGaN NWs were grown for 60 min immediately after the agglomeration of Ni. Figure S3 shows typical FE-SEM tilted images of the as-grown InGaN NWs as a function of the starting Ni film thickness. The results revealed that the morphology, density and degree of vertical alignment in the InGaN NWs were highly dependent on the size of the Ni catalysts. Dense and vertically aligned InGaN NWs were produced using the thinnest Ni film (0.3 nm), which produced the smallest Ni nanocatalysts with a density of approximately $3x10^3$ μm^{-2}. The overall density of the as-grown InGaN NWs at this film thickness was estimated, based on several top-view SEM images, to be approximately 8.4 μm^{-2}, where the density of vertically aligned InGaN NWs was approximately two times higher than that of tilted NWs. With increasing Ni film thickness, the density of InGaN NWs significantly decreased, and at 2 nm, growth virtually ceased. The observed morphological evolution of InGaN NWs as a function of Ni thickness can be explained through the change in the melting point of Ni nanocatalysts with respect to their size. For instance, it has been reported that the melting point of the agglomerated Ni nanocrystals is inversely proportional to their size [1]. In addition, the growth of NWs by metal-catalysed growth requires a high degree of supersaturation. Because small size Ni catalysts are expected to melt more easily, they can provide dense nucleation sites and facilitate the absorption of Ga and In adatoms during NW growth. Consequently, smaller Ni catalysts can reach the supersaturation stage faster than larger particles, and the growth of InGaN NWs with the latter can be partially limited by the high melting temperature.

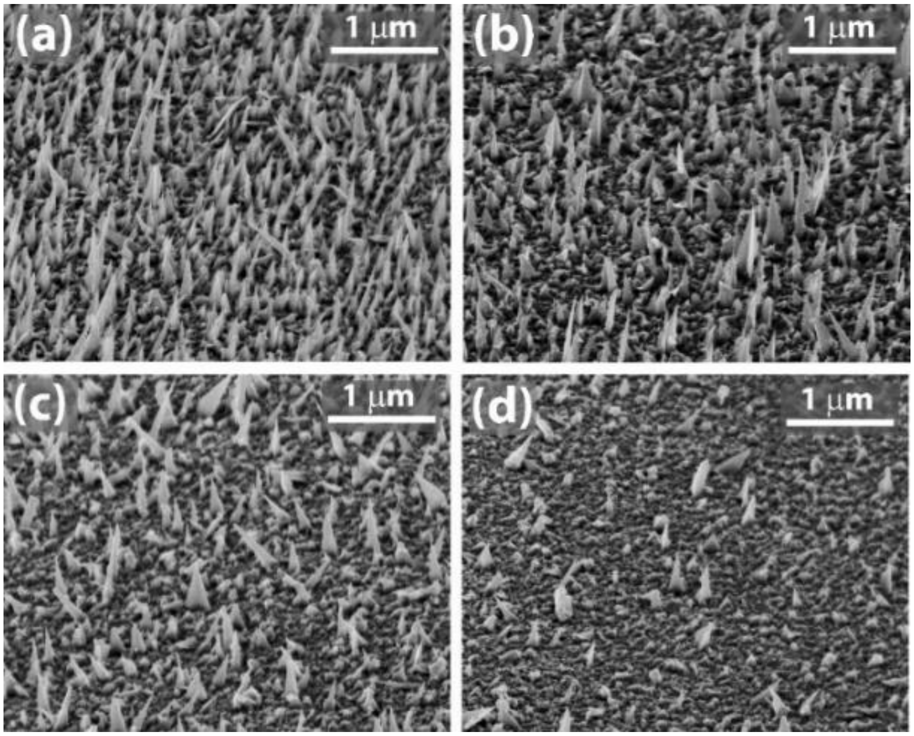

Figure S3. Typical FE-SEM images showing the influence of the Ni film thickness on the growth of InGaN NWs: (a) 0.3 nm; (b) 0.5 nm; (c) 1.0 nm; (d) 2.0 nm.

S4: *The variation of the PL peak energy as a function of temperature:*

The temperature-dependent PL spectra were normalized and are plotted in Fig. S4. The temperature-dependent variation of the PL peak energy displayed an S-shaped behaviour, which was prominent in the low energy peak (high In content). This S-shaped variation gradually decreased for InGaN samples grown under EIF and virtually disappeared in the EIF sample grown at 730 ˚C. This implies that the exciton localization effects should also follow the same trend.

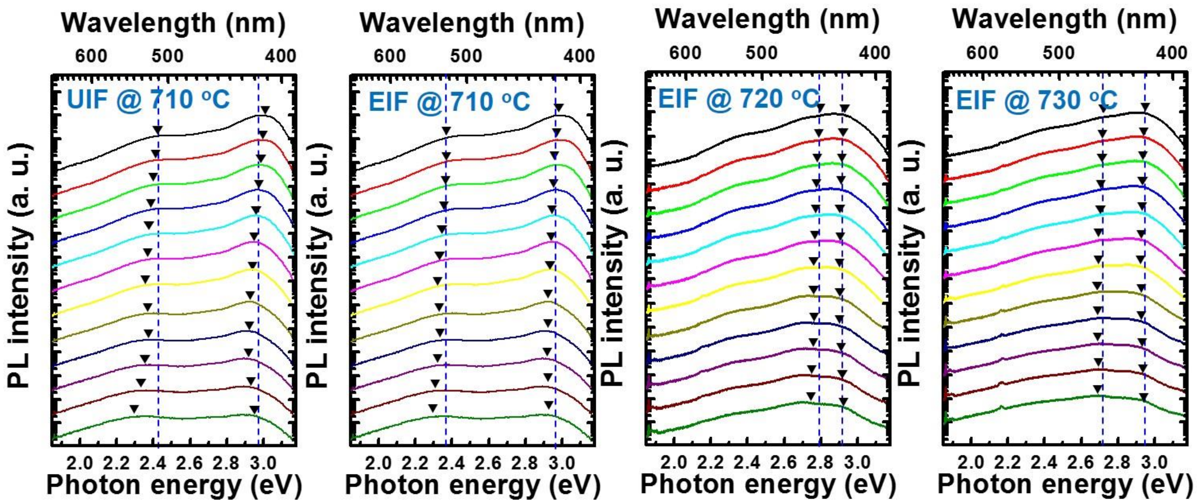

Figure S4. The PL peak energy variation with temperature measured by temperature-dependent PL for the UIF reference sample and the EIF samples.
